# Calcification of the Thoracic Aorta and Its Segments and Chronic Kidney Disease in Participants of the ELSA-Brasil Cohort

**DOI:** 10.1155/ijne/9818803

**Published:** 2025-11-02

**Authors:** Júlia Sosa Antunes Cândido, Luisa Campos Brant, Lidyane Valle Camelo, Jesiana Ferreira Pedrosa, Luana Giatti, José Geraldo Mill, Antonio Luiz Pinho Ribeiro, Sandhi Maria Barreto

**Affiliations:** ^1^Faculty of Medicine, Universidade Federal de Minas Gerais, Belo Horizonte, Minas Gerais, Brazil; ^2^Faculty of Medicine and Clinical Hospital/Ebserh, Universidade Federal de Minas Gerais, Belo Horizonte, Minas Gerais, Brazil; ^3^Universidade Federal do Espírito Santo, Vitória, Brazil

**Keywords:** arterial stiffness, calcification of thoracic aorta and segments, chronic kidney disease

## Abstract

**Introduction:**

Aortic calcification may be a vascular marker of health risk. Loss of elastic recoil due to arterial calcification results in hemodynamic changes that, in turn, can lead to damage to target organs, such as the kidneys. There are few studies analyzing the association between the presence of calcification in the thoracic aorta and chronic kidney disease (CKD).

**Objective:**

To investigate the association between calcification of transthoracic aortic (TAC) and its segments and CKD in individuals living in the community without established cardiovascular disease and to verify whether arterial stiffness is a confounder of this relationship.

**Methods:**

Cross-sectional study with 2427 participants from visit 2 of ELSA-Brasil, in Minas Gerais (2012–2015). TAC and its ascending (ATAC), aortic arch (AAC), and descending (DTAC) segments were categorized by the degree of calcification (0; greater than 0 and less than 100 HU; and greater than 100 HU). The presence of CKD was verified by glomerular filtration rate (eGFR CKDEPI) < 60 mL/min/1.73 m^2^ and/or albumin/creatinine ratio ≥ 30 mg/g. The adjustment covariates were age, sex, race/color, schooling, smoking, cholesterol/HDL ratio, BMI, diabetes, hypertension, and pulse wave velocity (PWV). Logistic regression models were performed to analyze the associations. Statistical significance was defined as *p* < 0.05.

**Results:**

After all adjustments, there was an association between DTAC and CKD in the group with the highest degree of calcification (OR: 2.66–1.05; 6.71). The inclusion of PWV in the final model slightly increased the magnitude of the association with DTAC (OR: 2.75; 1.07–7.05). No statistical association was found for TAC, ATAC, and AAC.

**Conclusion:**

Greater degree of DTAC is positively associated with CKD, regardless of the level of arterial stiffening.

## 1. Introduction

The investigation of subclinical atherosclerosis is an important challenge, allowing individuals at higher risk for cardiovascular events to be identified and providing better guidance for primary prevention strategies [1]. The elastic properties of the aorta contribute not only to supplying continuous blood flow but also to protecting sensitive organs from pulsatile pressure spikes. In fact, the loss of elastic recoil due to arterial calcification results in hemodynamic changes, such as an increase in systolic blood pressure (SBP), as well as in the compromise of target organs, such as the kidney [2]. The damage caused to the glomeruli in such conditions increases if renal autoregulation is impaired [3].

Despite this evidence, empirical studies that have directly analyzed the association between the presence of calcification in major arteries and chronic kidney disease (CKD) are still scarce [4]. The few available have mainly focused on the calcification of the thoracic aorta (TAC) and its segments. Moreover, they also were conducted in populations with previously diagnosed CKD [3, 5, 6]. Studies in populations living in the community are necessary to evaluate not only the role of calcification in the progression of CKD but also its contribution to the occurrence of CKD. This assessment is critical among those without established cardiovascular disease (CVD), since CVD is the main cause of arterial and renal damage. Furthermore, CVD is the leading cause of mortality in patients with CKD and this high mortality rate can be partially related by increased aortic calcification [7, 8].

It is not yet known whether the relationship between calcification of TAC and CKD depends on the calcification location according to its segments (descending and ascending aortic arch). This investigation is fundamental, as it is known that each aortic segment is susceptible to different hemodynamic stresses, which makes calcification throughout the aorta heterogeneous [9]. As calcification in each aortic segment appears to have different predictive values for cardiovascular and noncardiovascular morbidity and mortality [7, 10], it is possible that the association with CKD also varies according to the aortic segment affected by calcium deposits.

There is evidence that arterial stiffness is implicated in both the incidence of CKD [11–13] and the incidence of TAC [14, 15]. However, previous studies that have analyzed the association between the calcification of large arteries and CKD were not adjusted for arterial stiffness. This would be particularly relevant as arterial stiffness may be an important confounder of this association, as it is a common cause of both CKD and TAC.

This study aims to investigate the association between TAC and its segments and CKD in individuals in the community with no previously diagnosed CVD and to verify whether arterial stiffness is a confounder of this association. It is hypothesized that greater calcification in these sites is associated with a higher magnitude of association with chronic kidney disease (CKD), that the strength of this association varies according to the specific segment of the thoracic aorta, and that this relationship is independent of arterial stiffness.

## 2. Methods

This is a cross-sectional study including participants from the ELSA-Brasil cohort in Belo Horizonte, who underwent computed tomography (CT) to detect the presence of calcification in the thoracic aorta. ELSA-Brasil is a prospective multicenter study, developed with 15,105 public servants, aged from 35 to 74 years, recruited from higher education and research institutions, in six Brazilian capitals: Belo Horizonte, Porto Alegre, Rio de Janeiro, Salvador, São Paulo, and Vitória.

All 2923 participants in the second in-person follow-up visit of the ELSA-Brasil cohort (2012–2015) in Belo Horizonte, Minas Gerais, were invited to undergo CT to identify the presence of calcification in the coronary arteries, thoracic aorta, and carotid arteries in 2015-2016. The exclusion criteria for this examination were pregnancy, postpartum period, breastfeeding (up to 6 months postpartum), exposure to radiation at work, presence of metal in the chest, current radiotherapy, and nonparticipation in the second study visit. Multislice computed tomography (MSCT) was performed in 2638 individuals (90.2%). Participants with a history of acute myocardial infarction (*n* = 15), congestive heart failure (*n* = 21), stroke (*n* = 24), and/or cardiac surgery (*n* = 13) were excluded from the analysis. Furthermore, 142 participants underwent a different examination protocol, in which the aortic arch was not included. Thus, 2427 participants were included in the present study [10].

All cohort visit 2 participants underwent face-to-face interviews, clinical-epidemiological examinations, anthropometric measurements, and laboratory and imaging examinations with trained and certified research assistants.

ELSA-Brasil was approved by all Ethics Committees of the institutions involved in the study. All study participants signed the informed consent form before starting data collection at each visit.

### 2.1. Kidney Function Evaluation

On the second visit, creatinine was measured using the colorimetric enzymatic test (Jaffé method), and urinary albumin detection was measured using an immunochemical assay (nephelometry) [16]. Participants were instructed to collect urine every 12 h and underwent blood collection after fasting for 12 h [17]. The glomerular filtration rate (GFR) was estimated using the CKDEPI equation, without correction for race/color [18–20]. CKD was defined by GFR values below 60 mL/min/1.73 m^2^ and/or an albumin/creatinine ratio (ACR) greater than or equal to 30 mg/g.

### 2.2. Evaluation of Aortic Calcification

At visit 2, calcification measurements (Agatston score) in the total thoracic aorta, its ascending and descending segments, and the aortic arch were obtained. Participants underwent the same 64-slice MSCT scan (Lightspeed, General Electric, Chicago, IL, USA). The scanogram ranged from 1 cm above the top of the aortic arch to the apex of the heart. This method has been previously reported [21]. CT parameters were 2.5 mm thick slices with 20 × 0.62 mm collimation, 120 kVp, 100 mAs, and prospective electrocardiographic (ECG) triggering at 70% of the cardiac cycle. The reconstruction algorithm used a body filter, and the average calculated effective dose was 1.75 mSv.

The images were first analyzed by an experienced radiologist to identify the presence of calcifications in the thoracic aorta (TAC) as a whole. Next, for the present study, all images were reviewed by the radiologist, together with a qualified technician, to define the segments in which calcification were present. Finally, an interobserver and intraobserver correlation study was carried out with a random sample of 50 CT scans, which were scored twice by the radiologist and once by a second radiologist with 10 years of experience, resulting in intraclass correlation coefficients of greater than 0.99 for intraobserver and interobserver analyses [21].

**Figure 1:**
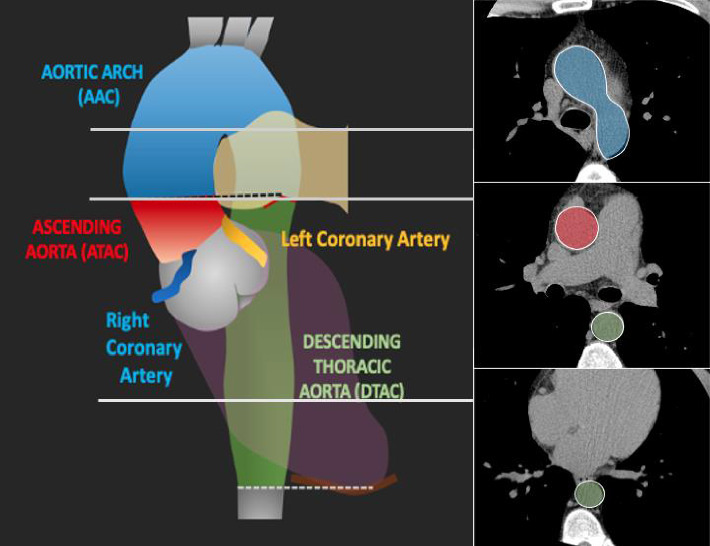
Anatomical references for thoracic aortic calcium score [21].

Calcium deposits were identified using semiautomatic software (Smart Score v4.0), which highlighted all calcium in green based on a threshold of 130 Hounsfield unit (HU) and calculated the Agatston score [22]. The observer reviewed each axial image and defined the calcium located in the arterial beds. In sum, calcium from the ascending thoracic aorta was considered from the sinotubular junction to the lower edge of the pulmonary artery bifurcation; therefore, calcium from the sinus of Valsalva and the aortic valve was not included. Descending thoracic aortic calcium (DTAC) was defined from the level of the lower border of the pulmonary artery bifurcation to the apex of the heart. Consequently, aortic arch calcium (AAC) was located above the ascending thoracic aortic calcium (ATAC) and DTAC using the same anatomical level as a reference (lower border of the pulmonary artery bifurcation) (Figure 1) [21].

Considering the degree of calcification, participants were classified into three categories (0; greater than 0 and less than 100 HU, and greater than 100 HU), created in order to enable a greater statistical power due to the small sample number with very high HU values, and because calcification with a density above 100 HU units is defined as a hyperattenuating lesion. In addition, these are the cutoff values used for coronary calcium score [22, 23]. Furthermore, for the ATAC analysis, only two groups were categorized (0 and greater than 0) due to the small sample size in the category with calcification greater than 100 HU and CKD (*n* = 7).

### 2.3. Covariables

Arterial stiffness was measured by pulse wave velocity (PWV), at baseline (2008–2010), obtained by a validated automatic device (Complior, Artech Medicale, France), with the participant lying down in a room with a temperature between 20°C and 24°C. PWV measures the stiffness of the aorta, a vascular territory of interest, as it is mainly responsible for the damping function of blood flow in the arterial bed and because it is an independent predictor of cardiovascular events in different populations [24]. Before measuring PWV, blood pressure was measured in the lying position with an oscillometric device (Omron HRM 705 CP) on the right arm. The distance from the sternal notch to the right femoral pulse was measured using a measuring tape. The pulse sensors were positioned in the right carotid and femoral arteries, allowing pulse waves to be viewed on a computer screen.

Software identifies pulse waves with good recording quality. PWV is calculated by dividing the distance from the wishbone to the femoral pulse by the time lag between the carotid and femoral pulses. The PWV of each participant was calculated by the arithmetic mean obtained in ten consecutive cardiac cycles at a regular heart rate. Carotid-to-femoral PWV records were recorded in all centers by certified research assistants and forwarded to a reading center, which was responsible for checking and excluding inappropriate exams [24]. PWV > 10 m/s was considered to pose a higher risk of target organ events, as previously demonstrated [25, 26].

The remaining covariates were collected at the second visit (2012–14). The following sociodemographic variables were considered: age, sex, self-reported race/color (white, brown, and black—indigenous and yellow individuals were excluded due to their low numbers in the sample), and schooling level (undergraduate studies, complete high school, and less than high school). Our study also considered the following behavioral variables: smoking and body mass index (BMI). The clinical variables were total cholesterol/HDL ratio, LDL cholesterol, diabetes mellitus (DM), and systemic arterial hypertension. Current smoking (no/yes) was evaluated as consumption of at least 100 cigarettes throughout life and current smoking. BMI was calculated as weight (kg) divided by the square of height (m^2^), according to standardized techniques.

Total cholesterol and HDL-C were obtained using standardized automated enzymatic colorimetric methods in blood samples collected after a 12-hour fasting. The presence of DM was defined by the report of a medical diagnosis of DM and/or the use of medication for DM and/or fasting blood glucose ≥ 126 mg/dL and/or a 75 g glucose tolerance test ≥ 200 mg/dL and/or glycated hemoglobin ≥ 6.5%. Participants were classified as having hypertension if their SBP was ≥ 140 mmHg, their diastolic blood pressure (DBP) was ≥ 90 mmHg, or they were using any medication to treat hypertension in the 2 weeks prior to the measurement.

### 2.4. Data Analysis

The total study population was described using mean and standard deviation for continuous variables and proportions for categorical variables. The same was done to describe the population's characteristics with calcification in the thoracic aorta and its segments. The ANOVA test was used for analysis between groups. Our study also described the prevalence of CKD according to GFR and ACR and the presence of TAC and segments. Likewise, in a supporting table, we also describe the distribution of the sample according to the presence of TAC and segments and the presence of increased arterial stiffness, defined as PWV > 10 m/s [25, 26].

The association between the total TAC categories and their segments and the presence of CKD was carried out using logistic regression, with the absence of CKD as the reference category. After performing the univariable analysis (Model 0) between the TAC categories and segments and CKD, our study added the following adjustment variables to the previous model: Model 0 + age (Model 1); Model 1 + sex, race/skin color, and education (Model 2); Model 2 + smoking, BMI, total cholesterol/HDL ratio, hypertension, and DM (Model 3); Model 3 + PWV (m/s) (Model 4).

The significance level used was 5%. Analyses were performed using Stata 14.0 software (Stata Corporation, College Station, United States).

## 3. Results

### 3.1. Characteristics of the Study Population and Prevalence of CKD

Table 1 describes the characteristics of the general population and of the subgroups by degree of calcification (0; greater than 0 and less than 100HU; and greater than 100HU) in the thoracic aorta and its segments. It can be observed that the mean age of the general sample was 55.6 ± 8.7 years and it increases as the degree of calcification increases on the aorta segments. The majority of participants in the overall sample had completed higher education (67%), a pattern that was also observed among those with vascular calcification. The mean PWV was 9.2 m/s (±1.8) in the general population, a lower value when compared with a higher degree of calcification.

The prevalence of CKD was equal to 9.9%, with this prevalence being higher (*p* < 0.001) in individuals who had a higher degree of calcification in the thoracic aorta and its segments. Of the variables included in Table 1, there was no statistical difference in the prevalence of calcification in the thoracic aorta and its segments according to sex and race/skin color at the level of *p* < 0.05. Table 1 shows statistically significant differences in age, schooling, smoking, BMI, LDL cholesterol levels, diabetes, hypertension, PWV, eGFR, and ACR across calcification levels in the aorta (*p* < 0.01) and in almost all aorta segments. Individuals with TAC ≥ 100 HU are older, less educated, have higher rates of smoking, diabetes, hypertension, and CKD, and show higher means of BMI, LDL cholesterol, PWV, eGFR, and ACR. For example, diabetes prevalence and hypertension prevalence are 26.9% and 26.3% for TAC ≥ 100 HU, versus 9.1% and 9.0% for low or no calcification; mean PWV is 10.4 m/s for high TAC compared to 8.7 m/s without calcification.

### 3.2. Renal Impairment According to Thoracic Aorta Calcification Burden and PWV Level

Table 2 shows the prevalence of CKD, decreased GFR, and elevated ACR across the same calcification strata used in Table 1. This analysis aimed to examine the gradient of renal impairment associated with increasing calcification burden.

As can be seen in Table 2, the higher the degree of TAC and in all segments, the higher the prevalence of CKD, as well as higher prevalence of low GFR (< 60 mL/min/1.73 m^2^) and increased ACR (> 30 mg/g) (Table 2). It was also seen that the higher the degree of TAC and in all segments, the higher the mean PWV values. Furthermore, higher prevalence of increased PWV (> 10 m/s) was found in higher degrees of TAC and in all segments (Supporting Table (available (here))).

### 3.3. Association of TAC Degree and Its Segment With CKD

**Figure 2:**
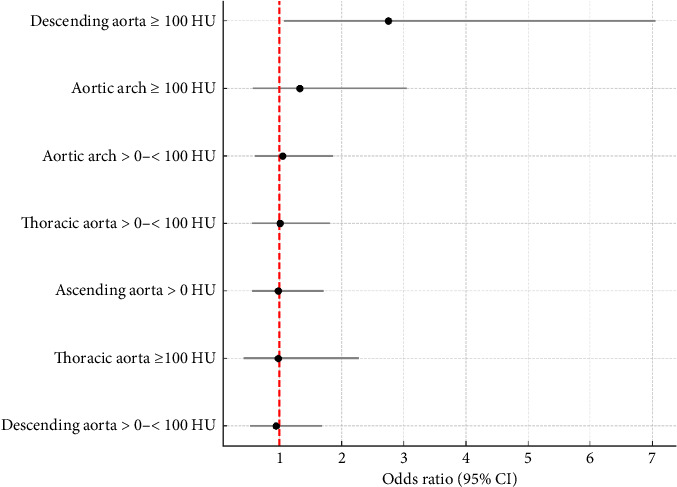
Forest plot illustrating odds ratios and 95% confidence intervals from Model 4, of the association between calcification of the thoracic aorta and segments and chronic kidney disease in ELSA-Brasil participants (2015-2016).

Table 3 displays the results from logistic regression models evaluating the association between thoracic aortic calcification and CKD. Models were progressively adjusted for sociodemographic factors, clinical risk factors, and PWV. To enhance visual interpretation of these results, a forest plot illustrating odds ratios and 95% confidence intervals from Model 4 was created (Figure 2).

In the univariate analysis, there was a positive association between the TAC and segments and CKD, in all calcification categories. However, when age was added as an adjustment variable, the association loses statistical significance, even in the category of greater volume of TAC (OR: 1.12; 0.71–1.76), of the ascending thoracic aorta (OR: 1.28; 0.95–1.71), and in the aortic arch (OR: 1.14; 0.74–1.77). These results did not change when the other variables were added. However, calcification in the descending thoracic aorta ≥ 100HU, but not > 0 and < 100HU, remained positively associated with CKD after adjustments for age (OR: 1.93; 1.23–3.04), with the strength of association proving to be even greater after the inclusion of the other confounding variables (OR: 2.66 (1.05; 6.71). The inclusion of PWV slightly increased the magnitude of this association (OR: 2.75; 1.07–7.05) (Table 3) (Figure 2).

## 4. Discussion

### 4.1. Main Findings and Segment-Specific Associations

To the best of our knowledge, this study is the first of its kind to investigate the relationship between TAC as a whole and each of its segments and CKD in individuals with no previously diagnosed CVD. Our study found that the presence of DTAC ≥ 100 HU increased the chance of CKD by 2.75-fold when compared to the group without calcification, but no association of calcification in a lesser degree was observed in this segment and CKD. Contrary to our hypothesis, no statistically significant relationship was found between TAC, ATAC, and AAC with CKD.

Some evidence may explain the association present only in those who have a higher degree of DTAC with CKD. Firstly, it is important to consider that vascular smooth muscle cells have a heterogeneous embryological origin, which can trigger different responses under calcification conditions, since the aortic arch derives from cardiac neural crest cells, while the descending aorta derives from the mesoderm [27]. Furthermore, each aortic segment is subject to different forms of hemodynamic stress, which also seems to affect the impact of calcification and its consequences [27]. In this light, studies have already found that the presence of calcification in different sites of the thoracic aorta is distinctly associated with cardiovascular risk factors and mortality, in addition to noncardiovascular morbidity and mortality, such as CKD, neoplasia, pneumonia, and dementia, among others [7, 28, 29].

Thus, our findings add to the study that found a relationship between DTAC and noncardiovascular outcomes, HR 1.06 (1.03–1.09), in 6765 individuals from the MESA cohort, with a mean age of 62 years, in 12 years of follow-up [7], suggesting that the presence of DTAC may be an early marker in those individuals without traditional or late-identified factors for CKD and CVD [7, 28]. DTAC is a common CT finding linked to systemic atherosclerosis, inflammation, and vascular events [30]. While not yet part of calcium scoring guidelines, it may help refine risk assessment [31]—especially when TAC is low or absent, in hypertension, or pre-op cardiac cases [32]. Our findings support the growing evidence that DTAC may reflect systemic atherosclerotic burden and vascular stiffness, both of which are associated with impaired renal function. From a clinical perspective, DTAC detected on routine chest CT imaging could serve as an accessible, noninvasive marker for early identification of patients at higher risk of CKD progression. Previous studies have shown that thoracic aortic calcification correlates with cardiovascular outcomes and all-cause mortality, suggesting that DTAC could be integrated into risk stratification algorithms in the future [23, 33]. Its use as a biomarker could guide preventive interventions, such as aggressive management of blood pressure, lipid control, or other vascular-related diseases, potentially delaying CKD progression and reducing cardiovascular risk. However, further prospective studies are needed to validate DTAC as an independent predictor of renal outcomes and to determine its incremental value over traditional risk factors.

Regarding the negative results with TAC, ATAC, and AAC, there are divergences in the literature. One previous study found that calcification of the aortic arch was associated with structural and functional changes in the renal vessels, which directly affect renal microcirculation, impairing the renal function in 568 individuals with stages 3–5 of CKD, defined by eGFR, based on the equation of the Modification of Diet in Renal Disease (MDRD) [33]. Another study, which also evaluated CKD patients in stages 3–5 (*n* = 237), also using MDRD, showed that calcification of the aortic arch was independently associated with a more accelerated decline in eGFR (*β* = −0.224), in 3 years, suggesting that calcification not only is a consequence of CKD but also predicts the progression of renal dysfunction [6]. Differences between the study populations may have influenced the divergence of these findings, since the aforementioned studies were restricted to individuals with previously diagnosed CKD, unlike the present study, which was carried out in a predominantly healthy population. Furthermore, discrepancies between results may also be due to the use of different GFR estimation equations, especially among older adults [34], as well as the noninclusion of ACR in the definition of CKD, since individuals with normal eGFR, but with albuminuria, tend to have a worse prognosis for CKD and CVD [35].

### 4.2. Vascular Mechanisms Underlying the Association With CKD

In the present study, carotid-to-femoral PWV was collected approximately 4 years prior to the evaluation of calcification in the aorta and segments; therefore, it is more likely that arterial stiffness contributed to the tomography findings than the other way around. It is believed that arterial stiffness remodels the structure of vascular wall, causing cell differentiation and mineralization, thus intensifying calcification in the vascular bed [30]. However, as this is a cross-sectional study, it is not possible to assess the direction of the relationship between PWV and calcification, although we know that vascular calcification induces the thickening of fibroelastic fiber and the reduction of elastic fibers, thereby contributing to greater arterial stiffening [15, 33]. There is consistent evidence that arterial stiffness predicts CKD [36–39]. Therefore, aortic stiffening evaluated by PWV may be both part of the mechanism underlying the association between vascular calcification and renal dysfunction [38], as well as a confounder of this relationship. In the present study, a clear relationship was identified between PWV and calcification in the different segments of the thoracic aorta (Supporting Table).

In this study, we also observed that the magnitude of the association between calcification in the descending thoracic aorta and CKD increased slightly after adjusting for PWV. Therefore, other factors seem to contribute to this association. One previous study found a direct relationship between atherosclerosis and renal deterioration [40], and aortic calcification is associated with atherosclerosis and stenotic lesions in the renal vascular system. Thus, in individuals with greater atherosclerosis, the decline in GFR could be a consequence of progressive renal hypoperfusion related to greater calcification in the descending thoracic aorta [3, 41].

### 4.3. Bidirectional Relationship Between Calcification and Kidney Dysfunction

Although the associations found between DTAC and CKD in this study corroborate evidence of associations between arterial calcification and CKD in previous longitudinal studies [6, 15], our results come from a cross-sectional study and therefore do not provide direct evidence of causality. It is also worth noting to mention that advanced CKD itself can accelerate arterial calcification, that is, there would be a bidirectional relationship between arterial calcification and renal dysfunction [4]. Deterioration of the renal function promotes increased arterial calcification due to the presence of risk factors for CVD or through hemodynamic and metabolic mechanisms, such as altered calcium and phosphorus metabolism, thereby leading to the calcification of the vessel walls [36, 42, 43]. However, it is important to highlight that, in our study, only 0.32% of the participants had an eGFR lower than 45 mL/min/1.73 m^2^, and the same number showed a highly altered ACR (≥ 300 mg/g), situations in which one can observe a higher effect of the renal function on vascular structural changes.

### 4.4. Study Limitations and Future Research Directions

The main limitation of the present study lies in its cross-sectional design making impossible to assess the direction of the associations between the variables studied. However, our results add an important contribution to the few studies that have analyzed the relationship between aortic calcification and CKD, especially TAC and segments, mainly in general populations without CVD and in the early stages of CKD, in addition to verifying that the relationship is not fully explained by presence of increased PWV. In the near future, ELSA-Brasil will make it possible to estimate the contribution between the bidirectional relationship between DTAC and PWV, as the latter measure was measured again in the third visit (after 9 years), as well as assess the longitudinal relationship between TAC and its segments and CKD.

## 5. Conclusion

Greater degree of DTAC was positively associated with CKD, in a relationship that appears to be independent of arterial stiffening. These results, if confirmed longitudinally, may help to anticipate the renal outcome and guide specific preventive measures for individuals with a high degree of calcification in this site.

## Figures and Tables

**Table 1:** Descriptive characteristics of the total study population and the population with thoracic aortic calcification and its segments among participants of the Belo Horizonte Adult Health Longitudinal Study (ELSA-Brasil, 2015-2016), free of cardiovascular disease (*N* = 2427).

Characteristics	Overall sample *n* = 2427 (100%)	Total thoracic aorta				Ascending thoracic aorta				Aortic arch				Descending thoracic aorta			
		TAC = 0 *N* = 712 (29.3%)	TAC > 0 & < 100 HU *N* = 1232 (50.7%)	TAC ≥ 100 HU *N* = 483 (20%)	*p* value	ATAC = 0 *N* = 1749 (72%)	ATAC > 0 & < 100 HU *N* = 650 (26.8%)	ATAC ≥ 100 HU *N* = 28 (1.1%)	*p* value	AAC = 0 *N* = 857 (35.3%)	AAC > 0 & < 1 00 HU *N* = 1190 (49%)	AAC ≥ 100 HU *N* = 380 (15.7%)	*p* value	DTAC = 0 *N* = 1542 (63.5%)	DTAC > 0 & < 100 HU*N* = 724 (29.8%)	DTAC ≥ 100 HU *N* = 161 (6.6%)	*p* value
Age (years)	55.6 (8.6)	50.2 (6.8)	55.6	63.3 (7.1)	**< 0.001**	53.8 (8.2)	59.8 (8)	67.2 (6.6)	0.300	50.8 (7.9)	56.4 (8)	63.7 (6.7)	**< 0.001**	53.1 (7.7)	58.6 (8.4)	65.4 (7.3)	**0.006**
Sex (%)					0.972				0.971				0.986				0.998
Female	54	53.5	56.1	49.7		53	56.9	57.1		54.2	55.5	49.2		54.9	54.3	45.3	
Race/color, (%)					0.272				0.83				0.437				0.137
White	50	48	48.8	56.6		49	52.1	70.8		48	49.8	55.6		49.3	48.6	65.1	
Brown	36.4	38.7	36.3	33		37.3	34.5	20.8		38.8	35.5	33.4		37.3	36.2	27.3	
Black	13.6	13.3	14.8	10.4		13.7	13.3	8.3		13.2	14.6	11		13.4	15.2	7.6	
Level of schooling, (%)					**< 0.001**				**< 0.001**				**< 0.001**				**< 0.001**
Undergraduate studies	67	73.1	65.9	60.6		68.7	62.8	57.1		72.7	65.0	60.4		70.1	61.3	63.3	
Complete high school	25.2	22.9	26.5	25.3		24.8	26.5	21.4		23.4	26.5	25.3		24.2	28.2	21.8	
Less than high school	7.7	3.9	7.4	14.1		6.7	10.6	21.4		3.9	8.5	14.2		5.7	10.5	14.9	
Smoking (%)	28.4	20.6	28.7	40.9	**< 0.001**	25.3	37.1	42.8	**< 0.001**	21.1	29.1	44.7	**< 0.001**	26.4	30.4	42.5	**< 0.001**
BMI (kg/m^2^)	26.8 (4.6)	25.8 (4.1)	27.2 (4.7)	27.5 (5)	**< 0.001**	26.3 (4.3)	28.4 (5.2)	27.2 (4.8)	**< 0.001**	25.9 (4.2)	27.3 (4.7)	27.5 (5)	**< 0.001**	26.2 (4.4)	28.2 (4.9)	26.8 (5.2)	**< 0.001**
Total cholesterol/HDL	3.7 (0.9)	3.6 (0.9)	3.8 (0.95)	3.7 (0.9)	0.492	3.7 (0.9)	3.8 (0.9)	4 (1.4)	**0.004**	3.7 (09)	3.8 (0.9)	3.7 (0.8)	0.379	3.7 (0.9)	3.8 (0.9)	3.8 (2.1)	0.187
LDL (mg/dL)	115.1 (13)	112.2 (28)	116.8 (28.7)	114.6 (36)	**0.015**	115 (29)	115.1 (30.2)	129.3 (78)	0.097	113.4 (28)	116.3 (29)	115.9 (37)	0.109	115.5 (29)	114 (31.7)	115.7 (30)	0.699
Diabetes mellitus, (%)	16	9.1	16	26.9	**< 0.001**	12.5	24.9	42.8	**< 0.001**	10	16.8	28.1	**< 0.001**	10.9	24	31	**< 0.001**
Hypertension (%)	13.7	9	14	26.3	**< 0.001**	11.6	20.8	33.3	**< 0.001**	9.3	15.3	25.8	**< 0.001**	11.7	16.6	38	**< 0.001**
PWV (m/s)	9.2 (1.7)	8.7 (1.3)	9.1 (1.5)	10.4 (2.3)	**< 0.001**	9 (1.6)	9.8 (2)	11.6 (2.6)	**< 0.001**	8.8 (1.4)	9.2 (1.6)	10.5 (2.3)	**< 0.001**	8.9 (1.4)	9.7 (1.7)	11.3 (2.8)	**< 0.001**
GFR (ml/min/1.73 m^2^)	88 (12)	90 (12)	87.3 (13)	84.3 (11)	**< 0.001**	88 (12)	86 (11)	84.2 (11)	**< 0.001**	90 (13)	87.4 (12)	84 (11.3)	**< 0.001**	89 (12)	86 (12.1)	84 (11.3)	**< 0.001**
ACR (mg/g)	7.3 (3.8)	6.8 (3.2)	7.4 (3.8)	7.9 (4.2)	**< 0.001**	7.1 (3.4)	8 (4.5)	9.9 (5)	**< 0.001**	6.9 (3.3)	7.4 (3.8)	8.1 (4.3)	**< 0.001**	7.1 (3.5)	7.7 (3.9)	8.5 (4.8)	**< 0.001**
CKD (%)	9.9	6.4	9.1	16.9	**< 0.001**	8	14.3	25	**< 0.001**	6.6	10	17	**< 0.001**	7.4	11.9	25.4	**< 0.001**

*Note:* The values in bold show which variables were statistically significant in the distribution according to the presence of calcification of the thoracic aorta and its segments.

Abbreviations: AAC, aortic arch calcification; ATAC, ascending thoracic aorta calcification; DTAC, descending thoracic aorta calcification; TAC, thoracic aorta calcification.

**Table 2:** Prevalence of chronic kidney disease (CKD), low glomerular filtration rate (GFR), and increased albumin/creatinine ratio (ACR), according to calcification in the thoracic aorta and its segments (ELSA, 2015-2016).

Calcification	CKD (%)	*p* value	GFR (< 60 mL/min/1.73 m^2^) (%)	*p* value	ACR (> 30 mg/g) (%)	*p* value
TAC		**< 0.001**		**< 0.001**		**< 0.001**
0	6.4		2.5		4.3	
> 0 & < 100 HU	9.2		5.7		4.5	
≥ 100 HU	16.9		10.8		8.4	
ATAC		**< 0.001**		**< 0.001**		**< 0.001**
0	8.0		4.9		4.0	
> 0 &< 100 HU	14.3		7.8		8.0	
≥ 100 HU	25		14.2		14.3	
AAC		**< 0.001**		**< 0.001**		**< 0.001**
0	6.6		2.9		4.2	
> 0 &< 100 HU	10		6.5		4.6	
≥ 100 HU	17.1		10		9.2	
DTAC		**< 0.001**		**< 0.001**		**< 0.001**
0	7.4		4.2		3.6	
> 0 &< 100 HU	11.8		7.0		6.5	
≥ 100 HU	25.4		14.9		14.2	

**Table 3:** Logistic regression models of the association between calcification of the thoracic aorta and segments and chronic kidney disease in ELSA-Brasil participants (2015-2016).

Degree of calcification	Thoracic aortic calcification OR (CI 95%)	Ascending thoracic aorta calcification^1^ OR (CI 95%)	Aortic arch calcification OR (CI 95%)	Descending thoracic aorta calcification OR (CI 95%)
Univariate model				
0	Ref	Ref	Ref	Ref
> 0 & < 100 HU	1.46 (1.02; 2.08)^∗^	1.97 (1.15; 2.59)^∗∗∗^	1.55 (1.12; 2.16)^∗∗^	1.68 (1.25; 2.26)^∗∗^
≥ 100 HU	2.96 (2.02; 4.33)^∗∗∗^	—	2.89 (1.98; 4.22)^∗∗∗^	4.27 (2.86; 6.40)^∗∗∗^
Model 1 + age				
0	Ref	Ref	Ref	Ref
> 0 & < 100 HU	0.95 (0.65; 1.39)	1.28 (0.95; 1.71)	1.01 (0.71; 1.44)	1.14 (0.83; 157)
≥ 100 HU	1.12 (0.71; 1.76)	—	1.14 (0.74; 1.77)	1.93 (1.23; 3.04)^∗∗^
Model 2—Model 1 + sex, race/color, and schooling				
0	Ref	Ref	Ref	Ref
> 0 & < 100 HU	1.07 (0.68; 1.67)	1.39 (0.96; 1.99)	1.05 (0.69; 1.60)	1.11 (0.75; 1.64)
≥ 100 HU	1.21 (0.69; 2.11)	—	1.25 (0.73; 2.16)	2.15 (1.21; 3.80)^∗∗^
Model 3—Model 2 + smoking, BMI, total cholesterol/HDL, hypertension, and DM				
0	Ref	Ref	Ref	Ref
> 0 & < 100 HU	1.02 (0.57; 1.82)	0.98 (0.56; 1.71)	1.05 (059; 1.86)	0.93 (0.52; 1.66)
≥ 100 HU	0.98 (0.42 2.25)	—	1.31 (0.57; 3.01)	2.66 (1.05; 6.71)^∗^
Model 4—Model 3 + PWV				
0	Ref	Ref	Ref	Ref
> 0 & < 100 HU	1.01 (0.56; 1.81)	0.98 (0.56; 1.71)	1.05 (0.60; 1.86)	0.94 (0.52; 1.68)
≥ 100 HU	0.98 (0.42; 2.27)	—	1.32 (0.57; 3.05)	2.75 (1.07; 7.05)^∗^

*Note:* The values in bold show which models were statistically significant, through logistic regression, based on the respective adjustments of each model. The *p* value is described in the legend.

Abbreviations: BMI, body mass index; CI, confidence interval; DM, diabetes mellitus; HU, Hounsfield units; OR, odds ratio; PWV, pulse wave velocity.

^1^Calcification level in the ascending thoracic aorta was categorized as 0 and > 0 due to very small number of individuals with ATAC > 100 HU.

^∗^ *p* < 0.5.

^∗∗^ *p* < 0.01.

^∗∗∗^ *p* < 0.001.

## Data Availability

Data are available on reasonable request. The data used in this study are available for research proposal on request to the ELSA's datacenter and to the ELSA's Publications Committee (publiELSA). Additional information can be obtained from the ELSA's datacenter (estatisticaelsa@ufrgs.br) and the ELSA Coordinator from the Research Center of Minas Gerais (sbarreto@medicina.ufmg.br).

## Supporting Information

Supporting Table: mean and standard deviation of pulse wave velocity (m/s) and prevalence of high pulse wave velocity (> 10 m/s), according to calcification in the thoracic aorta and according to segments (ELSA-Brasil, 2015-2016).
