# Supplementary material for: Calcification of the Thoracic Aorta and Its Segments and Chronic Kidney Disease in Participants of the ELSA-Brasil Cohort
Source: Int J Nephrol. 2025 Nov 2;2025:9818803. doi: 10.1155/ijne/9818803 (PMC12597233; doi:10.1155/ijne/9818803)
Supplement: Supporting Information — Additional supporting information can be found online in the Supporting Information section. [file 9818803.f1.docx]

| Supplementary Table. Mean and standard deviation of pulse wave velocity (m/s) and prevalence of high pulse wave velocity (>10 m/s), according to calcification in the thoracic aorta and according to segments (ELSA-Brasil, 2015-2016). | | |
| --- | --- | --- |
| **Calcification** | **PWV (m/s)**  **mean (SD)** | **PWV>10 m/s**  **(%)** |
|  |  |  |
| Thoracic aortic |  |  |
| *0* | 8.7 (1.3) | 14.9 |
| *>0 e <100UH* | 9.1 (1.5) | 23.4 |
| *≥100UH* | 10.4 (2.3) | 50.3 |
| Ascending thoracic aortic |  |  |
| *0* | 9.0 (1.6) | 21.7 |
| *>0 e <100UH* | 9.7 (2) | 36.6 |
| *≥100UH* | 11.6 (2.6) | 71.4 |
| Aortic arch |  |  |
| *0* | 8.8 (1.3) | 16.5 |
| *>0 e <100UH* | 9.2 (1.6) | 25.4 |
| *≥100UH* | 10.5 (2.3) | 51 |
| Descending thoracic aortic |  |  |
| *0* | 8.9 (1.4) | 17.5 |
| *>0 e <100UH* | 9.7 (1.7) | 35.7 |
| *≥100UH* | 11.3 (2.8) | 68.1 |
